# Coplanar versus noncoplanar intensity‐modulated radiation therapy (IMRT) and volumetric‐modulated arc therapy (VMAT) treatment planning for fronto‐temporal high‐grade glioma†

**DOI:** 10.1120/jacmp.v13i4.3826

**Published:** 2012-07-05

**Authors:** Valerie Panet‐Raymond, Will Ansbacher, Sergei Zavgorodni, Bill Bendorffe, Alan Nichol, Pauline T. Truong, Wayne Beckham, Maria Vlachaki

**Affiliations:** ^1^ Department of Radiation Oncology McGill University Health Centre Montreal QC; ^2^ Department of Radiation Oncology Vancouver Island Centre, BC Cancer Agency Victoria BC; ^3^ University of British Columbia Vancouver BC; ^4^ Department of Physics and Astronomy University of Victoria Victoria BC; ^5^ Department of Radiation Oncology Vancouver Centre, BC Cancer Agency Vancouver BC Canada

**Keywords:** volumetric‐modulated arc therapy (VMAT), intensity‐modulated radiation therapy (IMRT), high‐grade glioma

## Abstract

The purpose of this study was to compare dosimetric and radiobiological parameters of treatment plans using coplanar and noncoplanar beam arrangements in patients with fronto‐temporal high‐grade glioma (HGG) generated for intensity‐modulated radiotherapy (IMRT) or volumetric‐modulated arc therapy (VMAT). Ten cases of HGG overlapping the optic apparatus were selected. Four separate plans were created for each case: coplanar IMRT, noncoplanar IMRT (ncIMRT), VMAT, and noncoplanar VMAT (ncVMAT). The prescription dose was 60 Gy in 30 fractions. Dose‐volume histograms and equivalent uniform doses (EUD) for planning target volumes (PTVs) and organs at risk (OARs) were generated. The four techniques resulted in comparable mean, minimum, maximum PTV doses, and PTV EUDs (p≥0.33). The mean PTV dose and EUD averaged for all techniques were 59.98 Gy (Standard Deviation (SD)±0.15) and 59.86 Gy (SD±0.27). Noncoplanar IMRT significantly reduced contralateral anterior globe EUDs (6.7 Gy versus 8.2 Gy, p=0.05), while both ncIMRT and ncVMAT reduced contralateral retina EUDs (16 Gy versus 18.8 Gy, p=0.03). Noncoplanar techniques resulted in lower contralateral temporal lobe dose (22.2 Gy versus 24.7 Gy). Compared to IMRT, VMAT techniques required fewer monitor units (755 vs. 478, p≤0.001) but longer optimization times. Treatment delivery times were 6.1 and 10.5 minutes for coplanar and ncIMRT versus 2.9 and 5.0 minutes for coplanar and ncVMAT. In this study, all techniques achieved comparable target coverage. Superior sparing of contralateral optic structures was seen with ncIMRT. The VMAT techniques reduced treatment delivery duration but prolonged plan optimization times, compared to IMRT techniques. Technique selection should be individualized, based on patient‐specific clinical and dosimetric parameters.

PACS number: 87

## I. INTRODUCTION

As our understanding of the biology and pharmacogenetics of central nervous system (CNS) malignancies evolves, therapies have advanced to become more efficacious. In 2005, a randomized controlled trial in patients with glioblastoma multiforme (GBM) demonstrated a survival advantage to combining radiation therapy (RT) with concurrent and adjuvant temozolamide chemotherapy.^(^ ^1^ ^)^ Patients with a methylated O6‐methyl guanine methyltransferase (MGMT) promoter had a significant survival advantage over patients with a lack of MGMT methylation.^(^ ^2^ ^)^ Likewise, patients with grade III oligodendrogliomas, with a loss of heterozygosity at chromosomes 1p and 19q, have been shown to have a better prognosis with a median survival of several years.^(^ ^3^ ^)^ Thus, there are groups of patients with high‐grade brain tumors that can now be identified with prolonged survival. As survival outcomes improve, innovative methods to minimize treatment‐related, long‐term toxicities are needed to further improve the therapeutic ratio for patients with primary CNS tumors.

Clinical and dosimetric results of intensity‐modulated radiotherapy (IMRT) in high‐grade gliomas have been compared to traditional three‐dimensional conformal radiotherapy (3D CRT) techniques.^(^ ^4^ ^–^ ^7^ ^)^ IMRT demonstrated superior target dose conformality and homogeneity. In addition, IMRT decreased normal tissue doses, especially in cases where the target abutted critical structures such as the optic nerves and the brainstem.^(^ ^4^ ^–^ ^7^ ^)^ Others have shown the feasibility of using coplanar and noncoplanar IMRT techniques to treat patients with high‐grade gliomas, but data on direct dosimetric comparison between the two techniques are lacking.^(^ ^5^ ^,^ ^8^ ^–^ ^10^ ^)^ In a recent publication comparing the use of coplanar volumetric‐modulated arc therapy (VMAT) with coplanar fixed‐field IMRT, VMAT achieved equal or better target coverage, with improved sparing of normal organs at risk.^(^ ^11^ ^)^

The observation that coplanar techniques irradiate OARs such as the optic chiasm and optic nerves to adequately treat adjacent PTVs led to the hypothesis that techniques that involve noncoplanar beam arrangements may decrease doses to these same critical structures.^(^ ^11^ ^)^

As a result, this dosimetric study was conducted to compare four treatment planning methods: coplanar IMRT (IMRT), noncoplanar IMRT (ncIMRT), coplanar VMAT (VMAT), and noncoplanar VMAT (ncVMAT).

## II. MATERIALS AND METHODS

### A. Study subjects

Review and approval by appropriate review boards was secured prior to study initiation. Ten patients with high‐grade gliomas who had previously been treated with curative intent were selected. Case selection was limited to patients in whom the planning target volume (PTV) overlapped or was located within 3 mm of the optic apparatus.

### B. Treatment planning volumes

Patients were immobilized with a thermoplastic mask in the supine position followed by computed tomography (CT) scanning. The CT slices were acquired every 3 mm, exported to the study planning system (Eclipse; Varian Medical Systems, Palo Alto, California), and fused with the preoperative and postoperative magnetic resonance imaging (MRI) studies for gross tumor volume (GTV) delineation.

**Figure 1:**
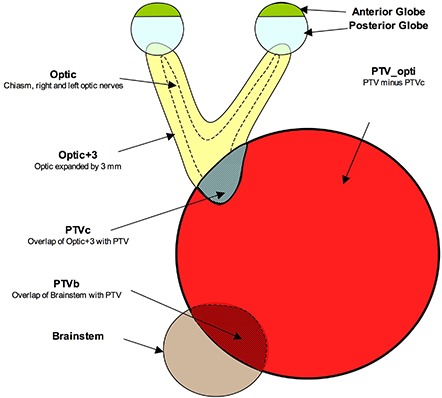
Diagram of the treatment planning volumes. Optic: includes the chiasm and both optic nerves; optic+3: optic expanded by 3 mm; PTVc: overlap of optic+3 and PTV; PTVb: overlap of brainstem and PTV; PTV_opti: PTV minus PTVc.

A consensus set of contours was generated by two radiation oncologists for the target, OARs and treatment volumes. A single plan technique was utilized in accordance with the European Organization for Research and Treatment of Cancer (EORTC) 26052_22053 protocol specifications. The GTV, and surgical bed, were outlined on the fused T1 contrast‐enhanced and T2 fluid attenuated inversion recovery (FLAIR) MRI images, expanded with a 2 cm margin to create the clinical target volume (CTV), then expanded by a further 0.5 cm to form the PTV. The following organs at risk were contoured and included in the analysis: ipsilateral and contralateral lenses, anterior globes (optical structures anterior to the most posterior extent of the lens), optic nerves, retinas, cochleas, temporal lobes, optic chiasm, brainstem, and uninvolved brain (brain – PTV). The lens was contoured as a separate structure with a 0.2 cm expansion to define the planning organs at risk volume (PRV). The optic chiasm and nerves were collectively expanded by a 0.3 cm margin into a structure known as Optic+3. No PRV was generated for any other OAR, even when it overlapped with PTV. A schematic of the representative planning volumes is depicted in Fig. 1. The overlap of Optic+3 with the PTV was defined as a target volume (PTVc) rather than an OAR, while Optic‐opti was derived after subtracting PTVc from Optic+3. PTVb was defined as the overlap of the brainstem with the PTV, and PTV_opti (optimized PTV), defined as PTV minus PTVc.

### C. Dose volume constraints

The PTV dose used for this study was 60 Gy in 30 fractions. The maximum dose permitted within the PTV was 110%. The minimum volume of the PTV covered by the 95% isodose line was 98%. Dose constraints are summarized in Table 1. The PTVc was limited to a maximum dose limit equal to 54 Gy (90% of the prescription dose) and a minimum dose limit of 51 Gy (85% of the prescription dose). Two additional target structures were used in plan optimization: PTVb, in which the maximum dose was limited to 60 Gy; and PTV_opti (optimized PTV).

**Table 1:** Dose constraints.

	*Max. Dose (Gy)*	*Constraint*
Optic_opti	54	$V_{50}\leq 50\%$
Brainstem	60	
Anterior Globe	30	
Retina	45	
Lens	10	
Contralateral Temporal Lobe		$V_{35}\leq 50\%$
Contralateral Cochlea	Mean dose Mean dose <45	
PTVc	$V_{50}<1\%$	51 Gy (min)
PTVb	60 (point dose)	$V_{58}\leq 15\%$
PTV	66	$V_{95}\geq 98$

PTVc: planning target volume (PTV) that overlaps with the optic structures planning organ at risk volume (Optic+3); PTVb: planning target volume that overlaps with the brainstem; Optic_opti: Optic+3 minus PTVc; max: maximum; min: minimum; Gy: Gray.

### D. Treatment planning

All plans were created specifically for the study. Planning was performed by an experienced dosimetrist. To minimize bias, cases selected at random were replanned by an experienced physicist with equivalent results. Plans were generated using an evaluation version of Eclipse (version 10.0.11). This system incorporated the anisotropic analytic algorithm (AAA) dose calculation algorithm. Four plans (IMRT and VMAT, both coplanar and noncoplanar) were generated for each of the ten cases.

The isocenter in each case was placed close to the center of gravity of the PTV. Planning commenced with conventional coplanar IMRT using approximately equally‐spaced entrance and exit gantry angles. These plans consisted of seven fields for midline PTVs and six fields for more lateral volumes. Where necessary, standard gantry angles were modified to exit rather than enter through the orbits. In addition, the collimator was rotated in cases where additional shielding of optic structures by the X and Y jaws was necessary.

Noncoplanar IMRT plans consisted of seven fields, including two to four noncoplanar fields. Couch rotations were limited to one or two angles. Coplanar VMAT plans consisted of a single arc field with the collimator at 45° and no avoidance sectors. Priorities and constraints were initially set to the values used in the final IMRT optimization. Modifications were made after each dose calculation, resulting in up to four iterations to achieve the end result. Noncoplanar VMAT plans also included a 180° arc at a couch angle of 90°. Depending on the location of the PTV, the starting gantry angle was as much as 20° superior–anterior, but more commonly at 0°.

Planning times were based on the time taken to complete the final optimization cycle. Volumetric dose calculation time was not included in the planning time as it was not felt to be representative of an actual clinical system with multiple calculation workstations. Treatment times were calculated and were determined from the mean dose rates for each field. An allowance for interfield gantry and couch rotations was included.

### E. Evaluation of treatment plans

The endpoints for the target included mean, minimum, and maximum PTV doses. It also included conformity index and homogeneity index. Conformity index was defined as the overlap of the fraction of the PTV enclosed by the 95% isodose (${\text{PTV}}_{95}$), with the fraction of the total body volume ($V_{95}$) covered by the same dose where:

(1) $$\text{CI}={\text{PTV}}_{95}{\;}^{2}/(V_{95}\cdot \text{PTV})$$

Homogeneity index (HI) was defined for the 95% and 105% isodose levels as:

(2) $$\text{HI}=1-{\text{PTV}}_{105}/{\text{PTV}}_{95}$$

The endpoints for the organs at risk included maximum and mean doses for each critical structure and the integral dose for the uninvolved brain. Cumulative DVHs were exported from the treatment planning system for each plan in the study, and the aforementioned indices were then calculated.

The EUD was calculated for both the PTV and OARs through a phenomenological expression, which represents a generalized mean dose, as proposed by Niemierko:^(^ ^12^ ^)^

(3) $$EUD={\left(\sum_{i}v_{i}D_{i}^{a}\right)}^{1/a}$$

where $v_{i}$ is a fractional volume receiving the dose $D_{i}$, and α is a model parameter. The values for this parameter were taken from Gay and Niemierko.^(^ ^13^ ^)^ As all plans were evaluated for 2 Gy per fraction treatments, no dose‐fractionation corrections were required.

### F. Data analysis

Results were compared by averaging specified endpoints over all patients for each treatment technique. Coplanar IMRT and VMAT plans were compared to noncoplanar IMRT and VMAT plans across the entire cohort using a two‐way analysis of variance (ANOVA). Analysis was performed using SPSS software with significance established at p<0.05.

## III. RESULTS

The four techniques resulted in comparable mean, minimum, and maximum PTV doses, and PTV EUDs (p≥0.33) (Table 2). Small but significant differences were observed in conformality indices (CI), with improved CI noted in VMAT plans (IMRT 0.88 and ncIMRT 0.89 versus VMAT 0.917 and ncVMAT 0.923, p<0.05), while homogeneity indices were similar across techniques evaluated (HI 0.99 for all techniques).

**Table 2:** Comparison of doses to PTVs and OARs in Gy.

	*IMRT*	*ncIMRT*	*VMAT*	*ncVMAT*
PTV mean	60.02	60.03	59.93	59.95
PTV max	66.15	66.15	66.45	66.50
Brain‐PTV mean	24.59	24.68	24.54	23.74
Brain‐PTV max	62.39	61.7	62.43	61.62
Brainstem mean	30.14	33.36	28.77	30.82
Brainstem max	56.30	56.42	56.35	56.74
Optic Chiasm mean	46.72	46.18	46.54	46.17
Optic Chiasm max	53.42	52.89	53.73	52.48
Ipsi Cochlea mean	53.89	39.52	35.84	38.38
Ipsi Cochlea max	40.12	42.96	40.55	43.00
Contra Cochlea mean	12.62	12.74	11.22	10.67
Contra Cochlea max	16.76	16.37	14.61	13.92

PTV: planning target volume; OAR: organs at risk; max: maximum; Gy: Gray; IMRT: coplanar intensity‐modulated radiotherapy; ncIMRT: noncoplanar intensity‐modulated radiotherapy; VMAT: coplanar volumetric‐modulated arc therapy; ncVMAT: noncoplanar volumetric‐modulated arc therapy; Ipsi: ipsilateral; Contra: contralateral.

Doses to the OAR, including the brainstem and uninvolved brain, were similar across techniques (Table 2). Specifically, IMRT, ncIMRT, VMAT, and ncVMAT resulted in mean doses to the uninvolved brain of 24.59, 24.68, 24.54, and 23.74 Gy, respectively. The volume of brain receiving 18 Gy and the integral brain dose were not significantly influenced by technique or beam arrangement. A trend for decreased dose to the contralateral temporal lobes was seen with noncoplanar beam arrangements with mean doses of 23.13 and 23.26 for ncIMRT and ncVMAT, compared to 26.86 and 26.88 for IMRT and VMAT.

**Figure 2:**
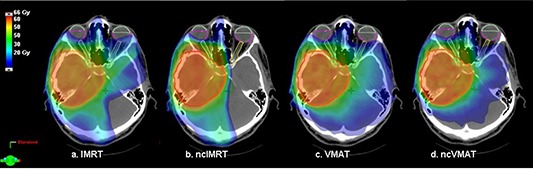
Single axial slices showing isodose distributions for: (a) coplanar intensity‐modulated radiotherapy (IMRT); (b) noncoplanar intensity‐modulated radiotherapy (ncIMRT); (c) coplanar volumetric‐modulated arc therapy (VMAT); (d) noncoplanar volumetric‐modulated arc therapy (ncVMAT).

**Figure 3:**
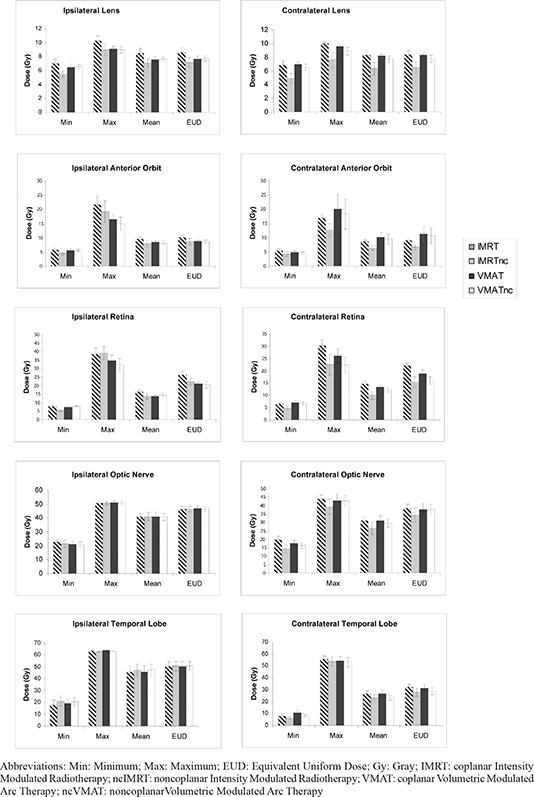
Abbreviations: Min: Minimum; Max: Maximum; EUD: Equivalent Uniform Dose; Gy: Gray; IMRT: coplanar Intensity Modulated Radiotherapy; ncIMRT: noncoplanar Intensity Modulated Radiotherapy; VMAT: coplanar Volumetric Modulated Arc Therapy; ncVMAT: noncoplanarVolumetric Modulated Arc Therapy Composite of minimum, maximum, mean, and equivalent uniform doses per technique for ipsilateral and contralateral optic structures and temporal lobes.

Noncoplanar IMRT displayed a consistent pattern of dose reduction to the contralateral optic structures, with a smaller comparative reduction in dose seen with ncVMAT (Figs. 2 and 3). Axial views of the isodose distributions in a representative case for each technique are depicted in Figs. 2(a)–2(d). Clear sparing of the contralateral orbital contents can be seen with ncIMRT (Fig. 2(b)). Specifically, ncIMRT significantly reduced doses to the contralateral retinas (p=0.041), and anterior globes (p=0.061) (Fig. 3). Noncoplanar IMRT resulted in significantly lower EUD to the contralateral anterior globe compared to IMRT, VMAT, and ncVMAT (7.20, 8.57, 7.64, and 7.74 Gy, p=0.05). In addition, both noncoplanar IMRT and VMAT significantly reduced contralateral retina EUDs (15.36 and 15.96 Gy) compared to IMRT and VMAT (22.05 and 18.79), (p=0.029). No significant differences between techniques were seen in mean doses or EUDs for ipsilateral optic structures, optic chiasm, brainstem or cochlea (Table 2).

Table 3 details the planning, optimizing, and treatment times for each technique. Compared to IMRT, VMAT techniques reduced the number of monitor units (MU) from 755 to 478 (p≤0.001). Plan optimization times increased with VMAT from 5 to 20 minutes. However, average treatment delivery times were significantly longer for IMRT and ncIMRT at 6.1 and 10.5 minutes, versus 2.9 and 5.0 minutes for coplanar and noncoplanar VMAT.

**Table 3:** Planning, optimization, and treatment times for each technique.

	*Mean Calculation*	*Mean Optimization*	*Mean Monitor*	*Mean Treatment*
*Technique*	*Time (min)*	*Time (min)*	*Units (range)*	*Time (min)*
IMRT	0.5	5	772 (584–937)	6.1
ncIMRT	0.5	5	737 (558–900)	10.5
VMAT	9.3	20	495 (381–788)	2.9
ncVMAT	10.4	20	461 (403–546)	5.0

IMRT: coplanar intensity‐modulated radiotherapy; ncIMRT: noncoplanar intensity‐modulated radiotherapy; VMAT: coplanar volumetric‐modulated arc therapy; ncVMAT: noncoplanar volumetric‐modulated arc therapy; min: minutes.

## IV. DISCUSSION

This study compared the dosimetry of coplanar and noncoplanar IMRT and VMAT planning techniques in cases of fronto‐temporal, high‐grade gliomas specifically selected for their challenging location abutting the optic apparatus. Our findings indicate that all four techniques result in clinically acceptable plans, with comparable target doses and dose to critical organs within prescribed dose constraints.

Our study explored the clinical implementation of novel VMAT planning capabilities: utilization of multiple noncoplanar arcs in VMAT planning and consistent use of inhomogeneity corrections in the dose calculation algorithm. Previous dosimetric studies have primarily focused on coplanar techniques.^(^ ^6^ ^,^ ^11^ ^)^ The utilization of multiple arcs in VMAT planning for high‐grade glioma has not been reported previously. Using a clinical system and a prerelease VMAT software, the current work highlights the feasibility of utilizing noncoplanar VMAT planning in a busy clinical setting, achieving realistic planning times.

In a study comparing volumetric single arc, helical arc, and fixed‐beam noncoplanar IMRT in benign brain tumor cases, Fogliata et al.^(^ ^14^ ^)^ demonstrated equivalent target coverage between techniques with $V_{95}>99\%$. In another study comparing IMRT to dynamic conformal arc in 25 cases of CNS tumors, Wiggenraad et al.^(^ ^15^ ^)^ reported that neither tumor type, size nor shape systematically predicted a preference for either technique. IMRT plan optimization times remain shorter than those seen with VMAT plans, but with recent innovations, both coplanar and noncoplanar VMAT planning can be considered feasible in a clinical setting. In addition, the use of two different dose calculation algorithms for VMAT and IMRT has further hindered direct plan comparison in the past. Our study allowed improved accuracy and consistency in plan comparison between techniques using the anisotropic analytic algorithm (AAA) throughout.

IMRT techniques resulted in significantly longer delivery times and an increase in monitor units when compared to VMAT. A longer delivery time impacts each daily fraction delivered, whereas a longer optimization time is only required initially in VMAT planning. As treatment times are compounded daily, the extra time and resources required to deliver certain techniques needs to be weighed against the benefit to the individual patient. Wagner et al.^(^ ^6^ ^)^ identified shorter treatment time, fewer MUs, and a small V107% as the major advantages when selecting RapidArc (Varian's version of VMAT) over IMRT in their comparative planning study of malignant glioma cases. In head‐and‐neck squamous cell carcinoma cases, VMAT plans required fewer MUs and shorter treatment times while maintaining similar dose distributions, compared to coplanar IMRT.^(^ ^16^ ^)^ When VMAT and IMRT were compared in left‐sided locoregional breast cancer radiotherapy planning, VMAT achieved similar PTV coverage and sparing of OAR, with fewer MUs and shorter delivery time.^(^ ^17^ ^)^

The effect of treatment delivery times on treatment effectiveness has been queried with *in vitro* evidence suggesting that the delivery of radiation in a compressed period of time improves cell kill.^(^ ^18^ ^,^ ^19^ ^)^ Furthermore, longer treatment times increase the potential for intrafraction motion and the dosimetric uncertainty that it can introduce. The impact of the longer IMRT plan treatment delivery times has yet to be clinically evaluated in comparison to VMAT plan delivery times but may be a consideration, especially for the relatively prolonged time necessary for ncIMRT delivery.

Noncoplanar IMRT resulted in superior sparing of contralateral optic structures with significantly lower anterior globe and retinal EUDs when compared to VMAT and coplanar IMRT. EUDs may be a surrogate for normal tissue complication probabilities for OARs. Sharma et al.^(^ ^20^ ^)^ compared noncoplanar conformal arcs with dynamic conformal arcs, IMRT and ncIMRT in four pediatric CNS cases. They concluded that ncIMRT resulted in OAR sparing in the largest and most complex shaped target. The sparing of the contralateral globe is unlikely to translate to a significant clinical advantage for most patients, but in patients with special circumstances such as unilateral blindness, individualized implementation of ncIMRT may confer an advantage.

IMRT has been previously shown to reduce the uninvolved brain volumes receiving 18 Gy when compared to 3D CRT.^(^ ^4^ ^)^ In our study, no significant differences were seen in mean and V18 doses to the brain with IMRT or VMAT or the use of noncoplanar beam arrangements. However, sparing of the contralateral temporal lobes was seen with noncoplanar techniques. Similarly, Sharma et al.^(^ ^20^ ^)^ noted better sparing of temporal lobes and uninvolved brain with ncIMRT when compared to noncoplanar conformal arcs, dynamic conformal arcs, and IMRT. With emerging evidence suggesting that temporal lobe sparing may translate into improved neurocognitive function preservation,^(^ ^21^ ^,^ ^22^ ^)^ future investigation into the use of noncoplanar beam arrangements, particularly in the pediatric population, is warranted. The need to achieve appropriate target coverage with high doses intermixed with delicate organ sparing is increasingly required in CNS planning strategy. This is especially true in pediatric cases where the expectations of long‐term survival and the need to minimize long‐term morbidity in the developing brain are essential practice considerations. While protons have been shown to produce optimal sparing,^(^ ^23^ ^)^ their limited availability preclude their widespread implementation. As such, evolving photon capabilities and technological innovations are being explored to maximize the therapeutic ratio in CNS malignancies, particularly in the pediatric subset. This may provide an additional setting in which further exploration of noncoplanar techniques and the sparing of structures such as the temporal lobes may confer a benefit. However, these benefits will need to be weighed against the increase in monitor units seen with ncIMRT plans.

## V. CONCLUSIONS

All techniques achieved comparable target coverage. Noncoplanar IMRT and VMAT techniques provided significantly better sparing of the contralateral optic structures than coplanar IMRT and VMAT techniques. VMAT techniques reduced treatment delivery duration but prolonged plan optimization times, compared to IMRT techniques. Technique selection should be individualized, based on patient‐specific clinical and dosimetric parameters.

## ACKNOWLEDGMENTS

Research funding support from a British Colombia Cancer Foundation Catalyst grant; planning software through an agreement with Varian Medical Systems
